# Supplementary material for: Dnmt1 has an essential function despite the absence of CpG DNA methylation in the red flour beetle Tribolium castaneum
Source: Sci Rep. 2018 Nov 7;8:16462. doi: 10.1038/s41598-018-34701-3 (PMC6220294; doi:10.1038/s41598-018-34701-3)
Supplement: Supplementary file 1 — Supplementary materials [file 41598_2018_34701_MOESM1_ESM.pdf]

Dnmt1 has an essential function despite the absence of CpG DNA methylation in the red flour beetle *Tribolium castaneum*

Nora KE Schulz, C Isabel Wagner, Julia Ebeling, Günter Raddatz, Maike F Diddens-de Buhr, Frank Lyko, Joachim Kurtz

Supplement

Table S 1 Primer sequences for RT qPCR (with efficiencies) and RNAi including information about size and location.

Gene	NCBI reference	Primer pair (5' – 3')	Fragment length (bp)	Location (start – end bp)	Efficiency	Primer origin
**RT qPCR**						
Dnmt1	XM_008195236	f: caagggcgagtgatacaccc<br>r: cgcagggctctcttgatct	185	Exon 11 – 12<br>(3520-3704)	1.89<br>(95%)	This study
rp49	XM_964471	f: ttatggcaaactcaaacgcaac<br>r: ggtagcatgtgcttcgttttg	132	Exon 2 – 3<br>(184-315)	1.98<br>(99%)	(1.)
rpl13a	XM_969211	f: ggccgcaagttctgtcac<br>r: ggtgaatggagccacttgtt	186	Exon 4 – 5<br>(488-673)	2.00<br>(100%)	(2.)
**RNAi**						
Dnmt1	XM_008195236	f: gcgagaaagtcttactgggg<br>r: cgcggctgaaattgcttagt	153	Exon 3<br>(542-694)	NA	This study
		f: acgacacgggcattttactc<br>r: tcatctctttcgcatcatcg	580	Exon 5-7<br>(1340-1918)	NA	iB _08496<br>(3.)
asnA	NC_000913.3	f: atggtggcggcaatacgtggatc<br>r: gattactccatcgcagaagctgc	304	442-768	NA	(4.)

1. Konopova B, Jindra M. *Proc. Nat. A. Sci*. USA. 2007;104:10488-93.
2. Peuß R et al. *Proc R Soc B*. 2015;282(1819).
3. obtained from EupheriaBiotech (http://2018.eupheria.net/)
4. Peuß R et al. *R. Soc. open sci*. 2016;3(160138).

Table S 2 RT qPCR temperature and cycle profile

Temperature	Time	Cycles
95°C	3 min	1
95°C<br>60°C	15 sec<br>1 min	40<br>(measuring fluorescence after each cycle)
95°C	continuous heating to obtain melting curve	
40°C	1 min	1
